# Nematode diversity in *Mastomys* rodents (Rodentia: Muridae) across a wildlife-human/domestic animal interface and molecular characterization of *Trichuris* species from *M. natalensis*

**DOI:** 10.1007/s00436-025-08507-y

**Published:** 2025-06-11

**Authors:** Jesse Mukisa Mutesasira, Sonja Matthee, Charles Byaruhanga, Milana Troskie, Munyaradzi Christopher Marufu

**Affiliations:** 1 Department of Veterinary Tropical Diseases, University of Pretoria, Private Bag X04, Onderstepoort, South Africa; 2 Department of Conservation Ecology and Entomology, Faculty of AgriSciences, Stellenbosch University, Stellenbosch, South Africa

**Keywords:** *Mastomys*, Nematode, *Trichuris*, Molecular characterization

## Abstract

Nematodes comprise many species with diverse life histories and zoonotic potential. Globally, over 10,500 nematode species were reported in mammals, particularly rodents. In South and southern Africa, data on nematode diversity in native rodent populations remains limited. The current study aimed to address this gap by reporting nematode diversity in two *Mastomys* species (*Mastomys coucha* and *M. natalensis*) and conducting molecular analysis of an unidentified *Trichuris* species recovered from *M. natalensis*. A total of 68 *M. natalensis* and 27 *M. coucha* rodents were trapped across a wildlife-human/domestic animal interface in South Africa during October 2020. Gastrointestinal nematodes were recovered and morphologically identified. Morphometric assessment and Sanger sequencing of mitochondrial markers, internal transcribed spacer 1 and 2 (ITS1, ITS2) and cytochrome b (*cytb*) were performed on the unidentified *Trichuris* species. In total, 46 nematodes were identified representing two genera, *Trichuris* and *Abbreviata*, with an overall prevalence of 21.1%. *Trichuris* sp. dominated in *M. natalensis* and anthropogenically transformed habitats, while *Abbreviata* sp. was associated with *M. coucha* in the natural habitats. Morphometrics of the unidentified *Trichuris* sp. aligned with reported ranges for the genus, while phylogenetic analyses revealed a novel clade distinct from known *Trichuris* spp. The current study emphasises the importance of integrating morphometric and molecular analyses for accurate nematode identification. The potential public health threat of the novel *Trichuris* sp. in *M. natalensis* needs further investigation, given the zoonotic capability of *Trichuris* spp. and the proximity of rodents to humans in the study area.

**Supplementary Information:**

The online version contains supplementary material available at10.1007/s00436-025-08507-y.

## Introduction

In Sub-Saharan Africa, *Mastomys* rodents are prolific agricultural pests (Mulungu 2017), and in South Africa they are known reservoirs of zoonotic pathogens, such as Lassa virus, *Yersinia pestis,* and *Bartonella* spp. (Singleton et al. 2003; Lecompte et al*.* 2006; Mhlanga et al. 2024). Eight *Mastomys* species have been reported across 30 African countries (Hánová et al. 2021). Of these, *M. natalensis* (Natal multimammate rat) is most widely spread in southern Africa (Calvet 2014) and semi-commensal*,* while* M. coucha* (southern multimammate mouse) is mainly found in South Africa, inhabiting both natural and anthropogenic habitats (Brouat et al. 2007; Monadjem et al. 2015; Little et al. 2024). *Mastomys* species are prolific breeders, live in family groups, and mainly eat seeds and arthropods (Mulungu et al. 2011; Mayamba et al. 2021). Both species thrive in anthropogenic habitats (Makundi and Massawe 2011; Bonwitt et al. 2017), attaining high population densities, and are associated with significant agricultural (Singleton et al. 2003; Prakash 2018) and public health challenges. In South Africa, the distribution of the two rodents overlaps in the north-eastern and eastern summer rainfall regions (Monadjem et al. 2015).

Despite their ecological and public health importance, complete understanding of nematode diversity within *Mastomys* rodents, particularly *M. natalensis* and *M. coucha*, is limited (Brouat et al. 2007; Jrijer et al. 2015; Julius et al. 2018a; Spickett et al. 2019a). Studies in Senegal (Brouat et al. 2007) and South Africa (Julius et al. 2018a; Spickett et al. 2019a) recorded nematode species such as *Heligmonina boomker*, *Neoheligmonella capensis*, *Trichuris muris*, *Syphacia obvelata*, and *Strongyloides ratti*, alongside at least seven unknown species in *M. natalensis* and *M. coucha* (Brouat et al. 2007; Julius et al. 2018a; Spickett et al. 2019b) highlighting an underestimation of the current nematode diversity. Limited research interest and the reliance on morphological identification, which is insufficient for taxa that include cryptic species, contribute to this underestimation.

The genus *Trichuris*, comprising several zoonotic species like *T. trichiura*, an important soil-transmitted helminth in humans (Brooker et al. 2004), *T. suis*, and *T. vulpis* (Taylor et al. 2001), is of particular interest due to its widespread presence in African rodents (Ribas et al. 2013). Morphological differentiation among *Trichuris* species is challenging, especially for species that occur in confamilial hosts (Feliu et al. 2000; García-Sánchez et al. 2019). Consequently, the use of molecular techniques has gained interest to differentiate between species and characterize potential new species (Ravasi et al. 2012; Callejon et al. 2017; Julius et al. 2018b; Rivero et al. 2020). Recent studies have identified several novel *Trichuris* species and unique genetic clades in African rodents using a combination of morphology and molecular analyses (Ribas et al. 2013, 2017). This study aimed to establish a better understanding of the diversity and distribution of nematodes of *M. natalensis* and *M. coucha* across a wildlife-human-domestic animal interface and to molecularly characterize the *Trichuris* sp. in *M. natalensis*.

## Materials and methods

### Study area

**Fig. 1:**
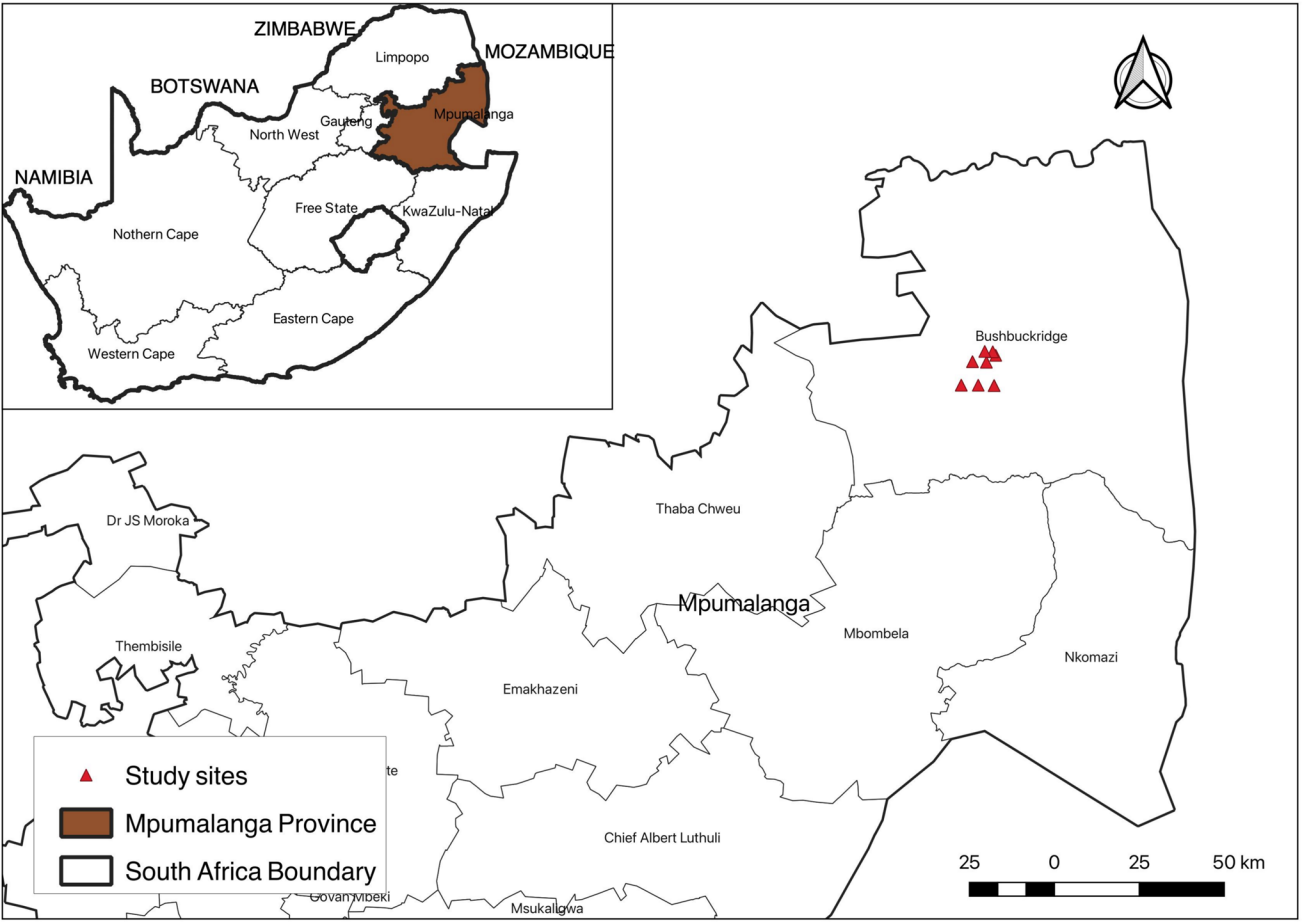
A map showing the localities for rodent sampling in the Mnisi community area, Mpumalanga Province, South Africa during October 2020. Insert (left) is a map of South Africa showing the location of Mpumalanga Province and the neighboring countries

Rodents were trapped in the Mnisi One Health Platform community, located in the northeastern savanna biome of the Bushbuckridge Municipal area, Mpumalanga Province, South Africa (Fig. 1). This biome is characterized by hot and wet summers and mild and cool winters. Average annual rainfall is 550 mm and average annual temperature is 21 °C (Murapa 2018). The area spans about 29,500 hectares of communal land, bordered by private and provincial conservation areas (Berrian et al. 2016). The Mnisi community primarily depends on livestock farming as the main agricultural activity and exhibits a strong interdependence between humans, domestic animals, and wildlife (David et al. 2013; Berrian et al. 2016). The study area is comprised of three habitats: crop, village, and natural reserves.

### Trapping and processing of rodents

Rodents were trapped using Sherman-type live traps following established procedures (Smith et al. 2023) during the austral spring of October 2020. Traps were set for four consecutive days at various sites in each of the three habitat types. To prevent trap-related mortality, traps were checked twice per day (10:00 and 15:00) and closed during the heat of the day. Target rodents were humanely euthanized (using isoflurane) and morphologically identified, first to genus level using reference guides (Skinner and Chimimba 2005; Monadjem et al. 2015) and then to species level by a species-specific multiplex PCR based on the cytochrome b region/gene, *cytb* (Bastos et al. 2005). The gastrointestinal tracts (GITs) were removed and preserved in 100% ethanol until examination.

### Nematode recovery and identification

The GIT of each rodent was placed in a petri dish containing 10 ml of physiologic saline solution and then split into four sections (stomach, small intestines, caecum, and colon) with the aid of a sterile scalpel and tissue forceps. Each section was transferred to a separate petri dish containing 10 ml of physiologic saline solution, gently teased open using a pair of curved Mayo dissecting scissors, a scalpel, and tissue forceps, and examined for helminths under a stereoscopic and light microscope. All nematodes recovered from each GIT section were rinsed with physiologic saline solution and fixed in 70% alcohol in labelled plastic tubes.

Recovered nematodes from all rodent specimens were microscopically identified to genus level following the guides by Thienpont et al. (1986). We then randomly selected *Mastomys* spp. from which more than one nematode had been recovered, and from each of these, seven individual nematodes of each morpho-species (*Trichuris* sp. from *M. natalensis* only and *Abbreviata* sp. from both *M. natalensis* and *M. coucha*) were slide-mounted in lactophenol for further morphological identification at the Agricultural Research Council-Onderstepoort Veterinary Institute (ARC-OVI) in Pretoria, South Africa, following taxonomic descriptions by various authors (Gasser and Monti 1997; Feliu et al. 2000; Khalil and Abdelmottaleb 2014). Specimens were also compared with reference material stored at the National Helminthological reference collection housed at ARC-OVI. Eight of the remaining ethanol-preserved *Trichuri*s specimens were kept for DNA extraction and PCR.

#### Morphological and morphometric measurements of nematodes

A total of three male and four female *Trichuris* specimens were randomly selected from *M. natalensis* individuals that had more than two *Trichuris* sp. specimens. A set of six morphological characters were measured for individual specimens. The characters were total body length, anterior body length, posterior body length, spicule length, spicule width at the base, vaginal length, egg length, and egg width to facilitate further identification. Visualization was done with an Olympus BX51 compound microscope fitted with an Olympus DP72 digital camera that was connected to a computer. Measurements were done using cellSens dimension software. All measurements were made in micrometers (µm) and subsequently converted to millimeters (mm) for ease of recording.

### DNA extraction from the nematodes

Eight ethanol-preserved *Trichuris* sp. specimens recovered from each of the *M. natalensis* rodents (*n* = 8) that had more than two *Trichuris* sp. specimens were used for DNA extraction and PCR. Only worms with complete body parts were selected for quality control since some were broken during the worm recovery process. Genomic DNA was extracted from whole-body tissue. First, each worm was placed in a 2-ml microcentrifuge tube containing three 3-mm borosilicate glass beads (Merck, Darmstadt, Germany) and 180 µl of buffer ATL (from the DNA extraction kit). The mixture was homogenized twice for 18 s at 5200 revolutions per minute (rpm) using a Precellys 24 homogenizer (Bertin Technologies, Montigny, France). The mixture was centrifuged at 18,064 g for 3 min and the supernatant aspirated for DNA extraction using the DNeasy Blood and Tissue Kit (QIAGEN, Hilden, Germany) according to the manufacturer’s instructions. The DNA was stored at − 20 °C until PCR analysis.

### PCR for Trichuris species

Three nematode-specific target regions or genes: ribosomal universal first internal transcribed spacer (ITS1), second internal transcribed spacer (ITS2), and *cytb* were selected for PCR amplification. The corresponding primer sets that target the genus *Trichuris* were as previously described (Table 3). The primers were synthesized and supplied by Inqaba Biotechnical Industries (Pty) Ltd. (Pretoria, South Africa). The ITS regions, although highly conserved, exhibit a high degree of sequence variation between different species, a characteristic of great importance for our phylogenetic analyses (Callejon et al. 2010). For each of the three regions/genes, the reaction mixture contained 12.5 µl of Phusion Flash High-Fidelity PCR Master Mix (Thermo Fisher Scientific, Johannesburg, South Africa), 0.5 µM each of the forward and reverse primers, 7.5 µl of nuclease-free water, and 2.5 µl of template DNA to make a total volume of 25 µl. Each PCR reaction was run in triplicate to ensure consistency and reliability of results by minimising the likely impact of stochastic amplification during PCR. In addition, this was done to average the random fluctuations and increase accuracy regarding the original DNA sample. A master mixture with a nuclease-free water template was used as a negative control. No reference sample was available, and therefore we did not include a positive control. The PCR conditions for amplification of each of the three target regions or genes started with an initial denaturation at 98 °C for 10 s, followed by 35 cycles of denaturation at 98 °C for 1 s, annealing, and extension (outlined below for each gene or region), a final extension stage at 72 °C for 1 min, and incubation at 4 °C. The annealing stage was at 55 °C for 1 min for the ITS1 region/gene, 55 °C for 30 s for the ITS2 region/gene, and at 50 °C for 30 s for the *cytb* region/gene. The extension stage was 72 °C for 8 s for ITS1 and ITS2 regions, and 72 °C for 6 s for the *cyt b.* The PCR products were visualized by agarose gel (2%, stained with ethidium bromide) electrophoresis at 120 V for 35 min.

### PCR purification, sequencing, and sequence analysis

The triplicate PCR products from each sample and for each of the three regions or genes were added in one tube to increase accuracy and reliability of the sequencing by minimizing the impact of stochastic amplification during PCR, given that the number of copies generated from a particular fragment varies randomly. This also reduces the impact of pipetting variations, which can introduce variability in the starting DNA material. The PCR product was then purified using the QIAquick PCR Purification Kit (QIAGEN, Hilden, Germany) following the manufacturer’s instructions. The purified PCR product was eluted in 50 µl of the elution buffer and then visualized by running on a 2% agarose gel (stained with ethidium bromide) at 120 V for 35 min alongside a 100-bp molecular marker (GeneRuler, ThermoScientific, Johannesburg, South Africa). About 10 µl of each purified PCR sample was submitted to the Central Analytical Facility of Stellenbosch University in Cape Town, South Africa for Sanger sequencing.

Sequencing of the purified amplicons was conducted using the respective reverse and forward primers for PCR amplification of each target region or gene. The AB1 files were quality trimmed, assembled, and cleaned using CLC Genomics Workbench version 7.5.1 (QIAGEN, Hilden, Germany). The Basic Local Alignment Search Tool (BLAST) (https://blast.ncbi.nlm.nih.gov/Blast.cgi) was used for taxonomic classification of the sequences and to retrieve homologous reference sequences. The obtained sequences of each region or gene, together with corresponding reference sequences, were aligned using Multiple Alignment using Fast Fourier Transform (MAFFT) software version 7 (Katoh and Standley 2013) and then visualized in BioEDIT version 7.2 (Hall 1999).

### Phylogenetic analysis

Phylogenetic trees were inferred using the maximum likelihood method, employing the General Time Reversible model with discrete gamma distribution and allowing for invariability for some sites (GTR + I + G). Analysis was done using the Molecular Evolutionary Genetics Analysis (MEGA) software version 11 (Tamura and Kumar 2021) with 1000 bootstrap replications. The genetic distances (proportion of nucleotide differences between pairs of sequences) were estimated using MEGA version 11 employing the p-distance method.

### Statistical analyses

Descriptive statistics were used to establish the proportion of infested rodents as well as median and mean helminth counts (overall, and for *Trichuris* sp. and *Abbreviata* sp.) from the host GITs. For the qualitative data, association between the presence of helminths (all worm species) and each of the predictor factors: habitat (crop, natural, village), rodent sex (male, female) and rodent species (*M*. *natalensis*, *M*. *coucha*) were analysed by binomial multivariable Generalised Linear Models. Data analyses were performed using the packages “doBy”, “pscl”, “MASS”, and “lmtest” in R statistical software version 4.2.1 (Rcoreteam 2022).

## Results

### Rodent and nematode abundances

Of the 95 *Mastomys* individuals examined, 56 were male and 39 were female. Most of the rodents were *M. natalensis* (*n* = 68) and the rest were *M. coucha* (*n* = 27).

Overall, nematodes were present in 21.1% (20/95) of the *Mastomys* individuals. A total of 46 nematode individuals representing two genera, *Trichuris* (*n* = 31) and *Abbreviata* (*n* = 15), were recorded. *Trichuris* sp. occurred in 11.6% (*n* = 11) of the rodents and *Abbreviata* sp. in 8.4% (*n* = 8). Single species infections were more common, with only one rodent harbouring both nematode species (Table 1). *Trichuris* sp. was primarily recorded in the caecum, while *Abbreviata* sp. was recorded in the stomach. *Trichuris* sp. was more prevalent in *M. natalensis* (17.6%), while *Abbreviata* sp. was more prevalent in *M. coucha* (22.2%). *Trichuris* sp. was recorded in all three habitat types, with the highest prevalence in the village (15.6%) followed by crop (11.5%) (Table 1). *Abbreviata* sp. was most prevalent in the natural habitat (20.8%) and was absent in the crop habitat.

**Table 1:** Nematode abundance and frequency of infection in *Mastomys* species (*n* = 95) categorized by habitat type, rodent sex and species. Rodents were captured across a wildlife-human-domestic animal interface in Mpumalanga Province, South Africa in October 2020

Variable	No. of rodents examined	Overall mean abundance ± SE, (frequency, %)	*Mastomys* with only *Trichuris* sp.: Mean abundance ± SE, (frequency, %)	*Mastomys* with only *Abbreviata* sp.: Mean abundance ± SE, (frequency, %)	*Mastomys* with mixed infections: Mean abundance ± SE, (frequency, %)
Habitat type					
Village	45	0.51 ± 0.34 (9.47)	0.44 ± 0.41 (15.56)	0.07 ± 0.15 (4.44)	-
Crop	26	0.42 ± 0.36 (5.26)	0.35 ± 0.33 (11.54)	-	0.19 ± 0.30 (3.85)
Natural	24	0.50 ± 0.34 (6.32)	0.04 ± 0.08 (4.17)	0.46 ± 0.37 (20.83)	-
Rodent species					
* M. natalensis*	68	0.49 ± 0.35 (19.12)	0.44 ± 0.36 (17.64)	0.03 ± 0.13 (1.47)	-
* M. coucha*	27	0.48 ± 0.33 (25.93)	0.04 ± 0.08 (3.70)	0.44 ± 0.35 (22.22)	-
Sex					
Male	60	0.12 ± 0.82 (20.00)	0.12 ± 0.57 (11.92)	0.07 ± 0.00 (6.83)	0.02 ± 0.50 (1.71)
Female	40	0.08 ± 1.00 (20.00)	0.10 ± 0.75 (10.00)	0.10 ± 0.00 (10.00)	-

*SE* standard error of mean

^a^A variation in rodent numbers is because the identity of five Mastomys specimens were not confirmed by nucleic acid-based analysis, and on dissection, they did not contain any nematodes

### Nematode abundance and association between nematode infections (presence) and predictor variables

Nematode abundances per individual rodent were in general low (< 5 on average, minimum = 0, maximum = 5, median = 1.5), and there was no statistically significant association (*p* > 0.05) between the presence (all worm species) of nematodes and each of the three predictor factors: habitat, rodent sex, and rodent species.

### Morphological features and morphometrics of nematodes

**Fig. 2:**
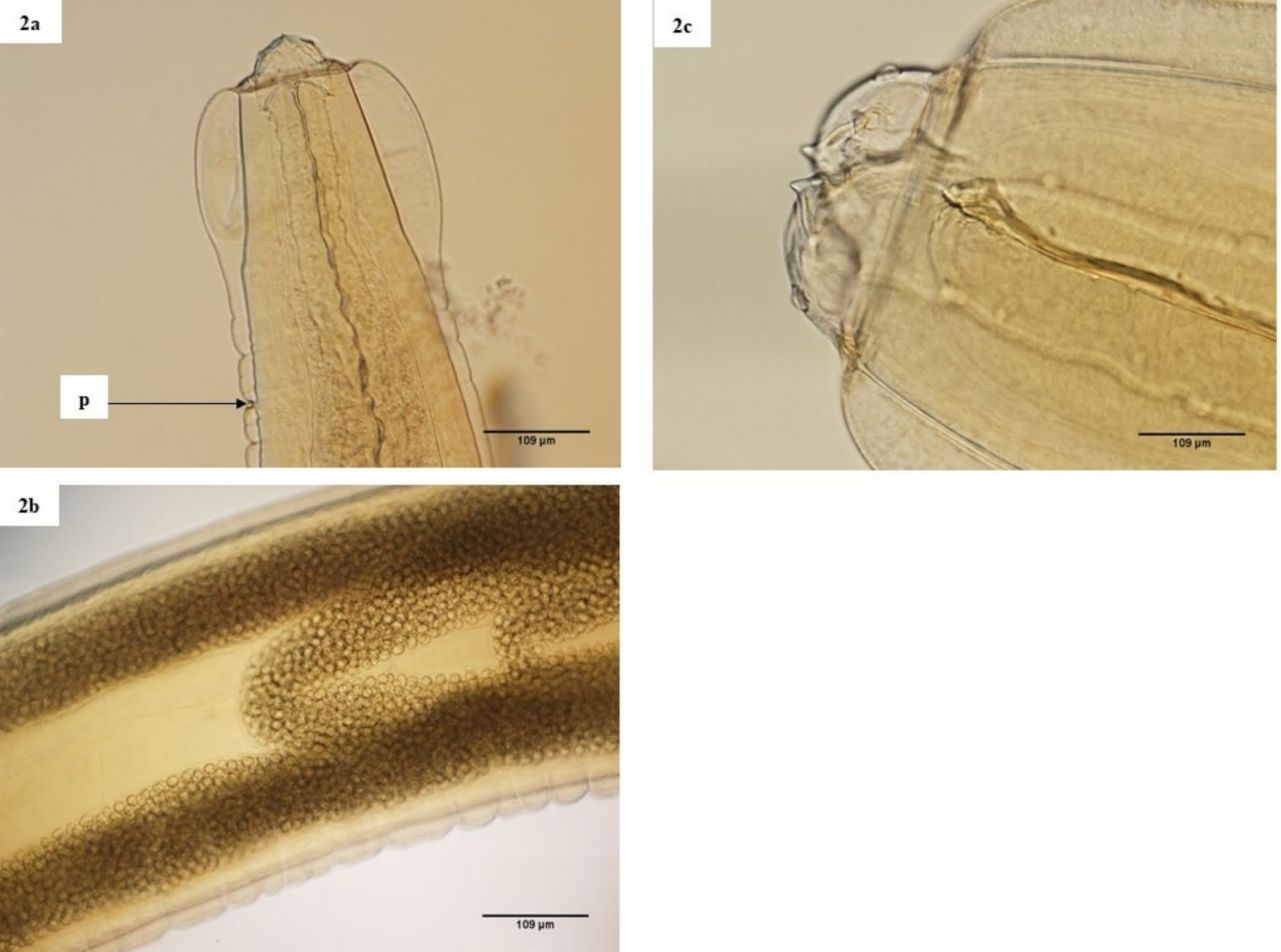
**a**–**c**: **a** Anterior end of *Abbreviata *sp. showing a laterally located excretory pore. **b** Characteristic uterine horns engorged with round eggs and **c** anterior end showing prominent bifid teeth

**Fig. 3:**
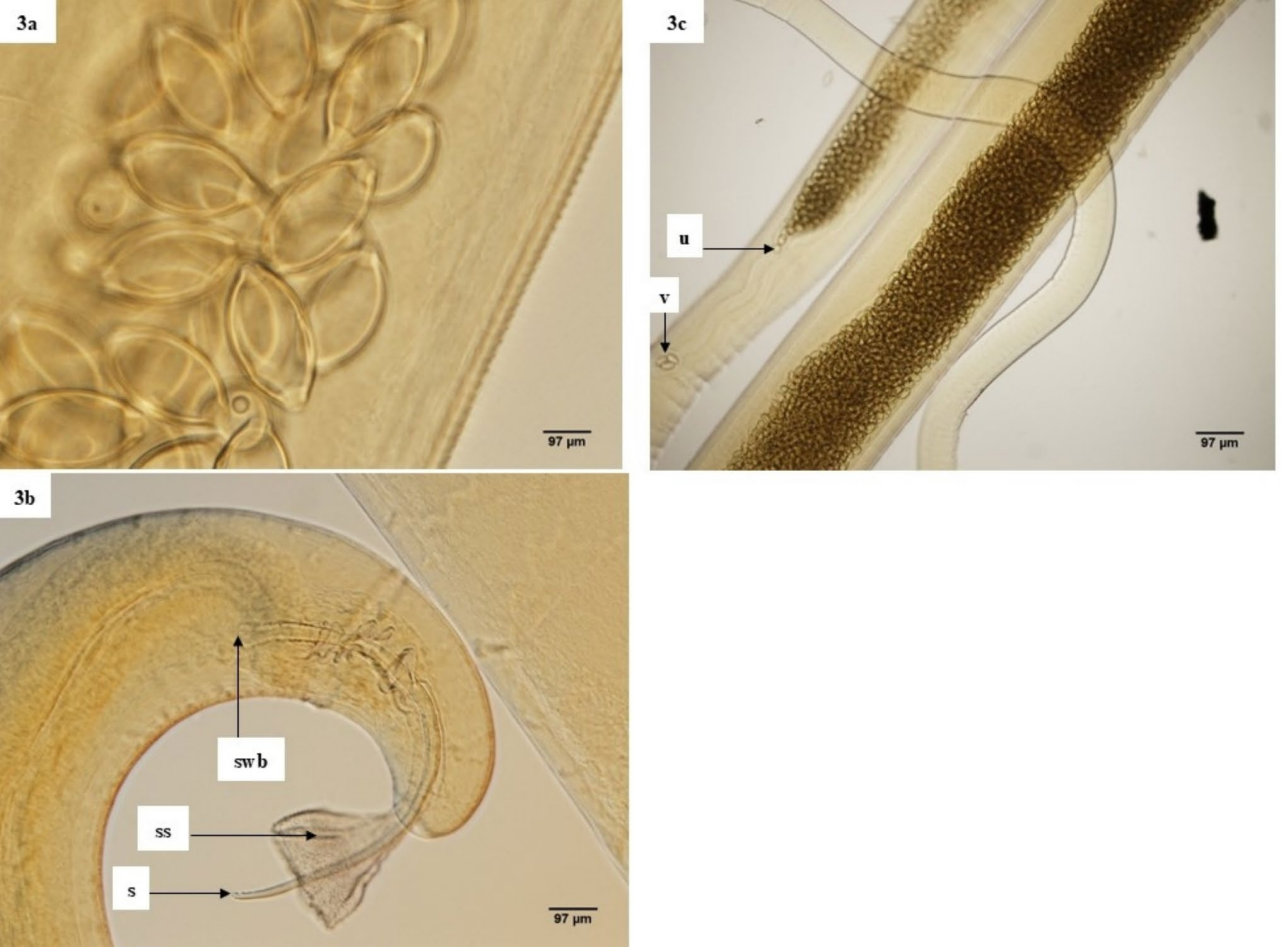
**a**–**c**: **a** Bi operculated ovoid eggs of *Trichuri*s sp. **b** Posterior end of male spicule of *Trichuris* species showing a spicule (s) covered by a spicule sheath (ss) and a spicule width at base (swb). **c** Middle part of the body of *Trichuris* species showing the vagina with eggs (v), beginning of the main body of an engorged uterus (u)

The morphological features of *Abbreviata* spp. as seen under the light microscope are shown in Fig. 2 with characteristic uterine folds (Fig. 2b). The average total body length in male *Trichuris* individuals was 21.80 mm, while the average anterior length was 15.16 mm, and the average posterior length was 7.64 mm. The average spicule length was 0.83 mm, and the average spicule width at the base was 0.04 mm (Table 2), with the spicule surrounded by an ornamented sheath (Fig. 3b). The average body length in females was 27.80 mm, while the average anterior length was 15.35 mm, and the average posterior length was 13.93 mm. The average vaginal length was 0.15 mm, and the average egg length was 0.06 mm. The average egg width was 0.030 mm, and the eggs had the characteristic ovoid and bi-operculation appearance (Fig. 3a and c).

**Table 2:** Morphometric measurements (in millimeter) for *Trichuris* species (3 males and 4 females) for selected characteristics. The nematodes were recovered from *Mastomys natalensis* across a wildlife-human-domestic animal interface in Mpumalanga Province, South Africa in October 2020

Sex	Character	Mean	Minimum–maximum	Median	Standard deviation
Male (*n*** = **3)	Total body length	21.80	18.11–25.49	21.80	3.69
	Anterior length	15.16	12.91–17.4	15.16	2.25
	Posterior length	7.64	5.20–9.60	6.65	1.45
	Spicule length	0.83	0.8–0.88	0.85	0.04
	Spicule width at base	0.04	0.037–0.04	0.039	0.0015
Female (*n*** = **4)	Total body length	27.80	22.60–33.00	27.80	5.20
	Anterior length	15.35	12.20–18.50	15.35	3.15
	Posterior length	13.93	10.40–16.50	14.40	2.20
	Vaginal length	0.15	0.13–0.16	0.154	0.01
	Egg length	0.06	0.06–0.06	0.06	0.0013
	Egg width	0.030	0.03–0.03	0.030	0.0008

### Sequence analysis

Eight sequence sets (forward and reverse) were obtained from the eight nematode specimens amplified for the ITS1 and ITS2 gene regions, but only seven sets for the *cytb* region/gene. The obtained sequence lengths were 571 to 582 bp for the *cytb* region/gene, 527 to 564 bp for the ITS1 region, and 548 to 589 bp for the ITS2. The BLASTn assessment showed that the identities of our sequences to published sequences were as follows: the ITS1 sequences were 80.4 to 80.5% identical to *T. muris* sequences (accession number FN543136; query cover 86%) from a murid host in Europe and 82.4% (query cover 67.0%) to *Trichuris* sp. Guinea from *M. natalensis* in Guinea (KX669078), while the ITS2 sequences were 78.9% to 82.5% identical (query cover 72.0%) to a *T. muris* isolate (KU575090) from a Muridae host in Europe and 82.4 to 83.5% identical to *Trichuris* sp. Benin from *M*. *natalensis* (KX669082). The *cytb* sequences were 83.9% and 86.5% identical (query cover 85.0%) to *Trichuris muris* isolate (LM994701) and 83.8% to 84.0% identical (query cover 99.0%) to *Trichuris* sp. ETH232 (MZ229688) from a Mahomet mouse in Ethiopia.

### Phylogenetic analysis

**Fig. 4:**
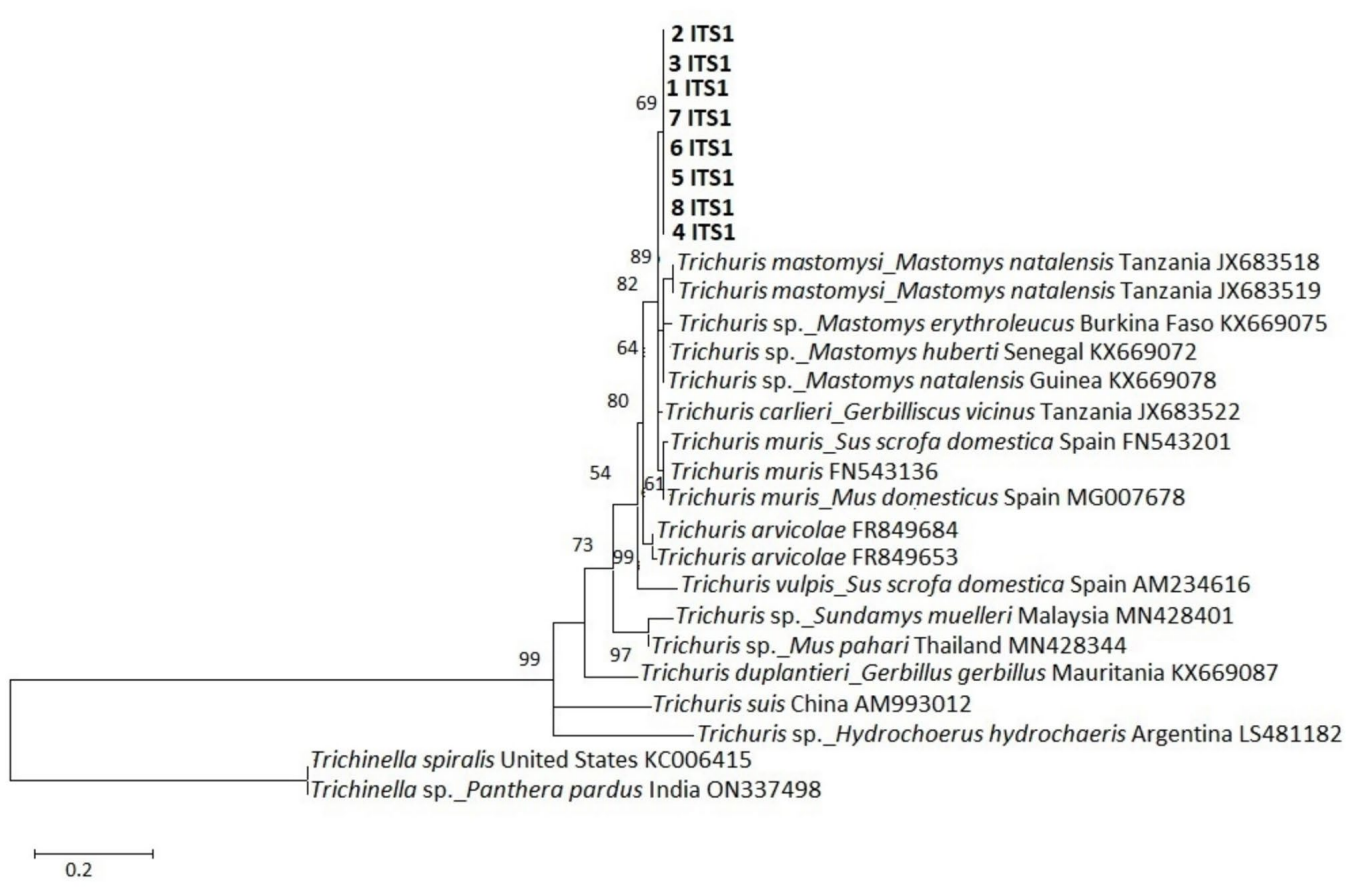
Phylogenetic analysis of *Trichuris* spp. ITS1 sequences obtained in this study together with published sequences. The tree was generated using a maximum likelihood method with 1000 bootstrap replicates as implemented in the Molecular Evolutionary Genetics Analysis (MEGA) software version 11.0. Scores at the nodes represent corresponding bootstrap support. The scale bar is proportional to the genetic distance in terms of nucleotide substitutions per site. The ITS1 sequences obtained in present study are highlighted in bold (1 ITS1 to 8 ITS1). The tree was rooted using a *Trichinella spiralis* sequence from the USA (GenBank accession number KC006415) and a *Trichinella* sp*.* sequence from a leopard (*Panthera pardus*) in India (ON337498). The *Trichuris* specimens were isolated from *M. natalensis* across a wildlife-human-domestic animal interface in Mpumalanga Province, South Africa in October 2020

**Fig. 5:**
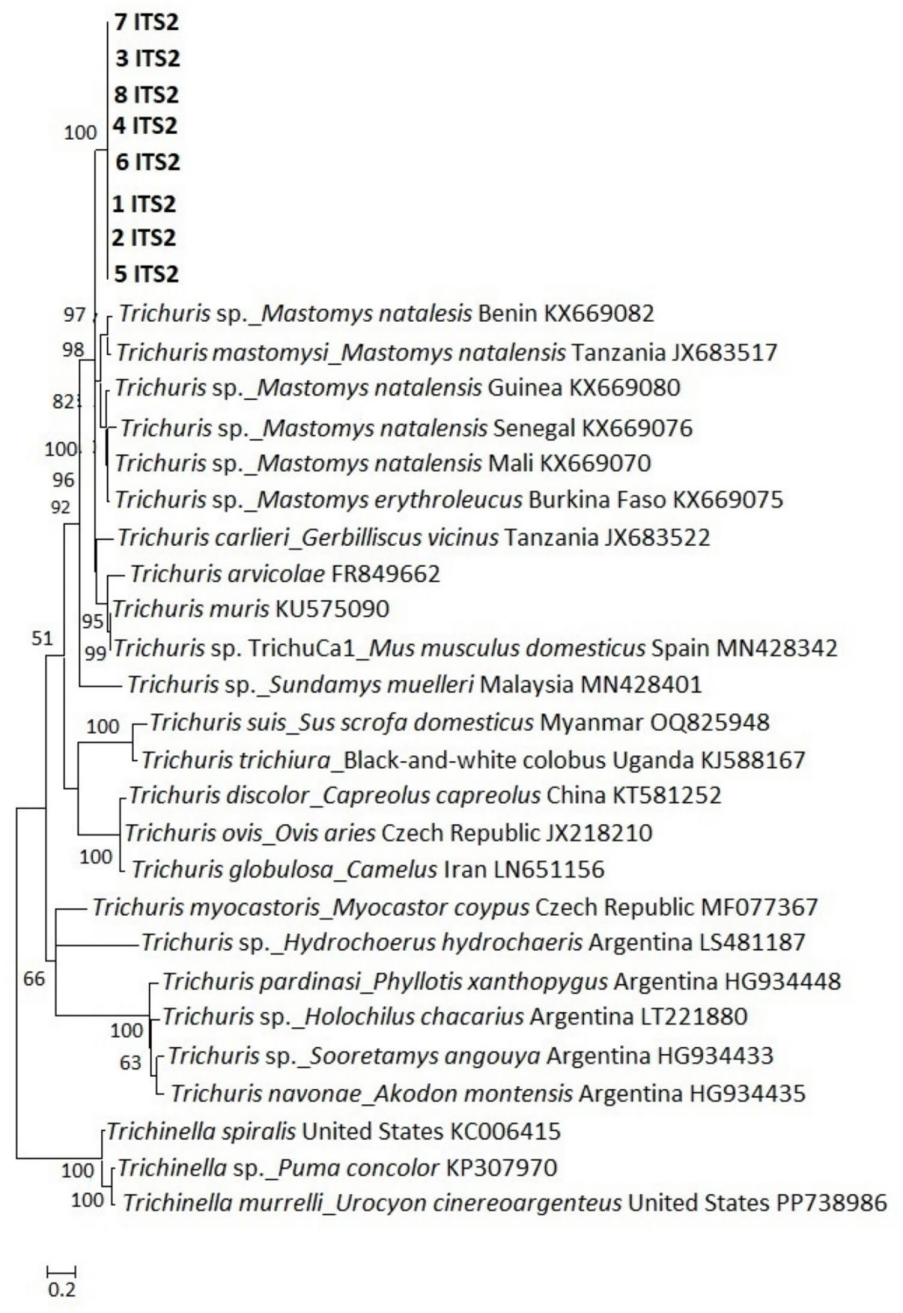
Phylogenetic analysis of *Trichuris* spp. ITS2 sequences obtained in this study together with published sequences. The tree was generated using a maximum likelihood method with 1000 bootstrap replicates as implemented in the Molecular Evolutionary Genetics Analysis (MEGA) software version 11.0. Scores at the nodes represent corresponding bootstrap support. The scale bar is proportional to the genetic distance in terms of nucleotide substitutions per site. The ITS2 sequences obtained in present study are highlighted in bold 1 ITS2 to 8 ITS2. The tree was rooted using a *Trichinella spiralis* sequence from the USA with GenBank accession number KC006415, *Trichinella* sp. sequence from cougar (*Puma concolor*, accession number KP307970) and a *Trichinella murrelli* sequence from gray fox (*Urocyon cinereoargenteus*) in the USA (accession number PP738986). The *Trichuris* specimens were isolated from *M. natalensis* across a wildlife-human-domestic animal interface, in Mpumalanga Province, South Africa in October 2020

**Fig. 6:**
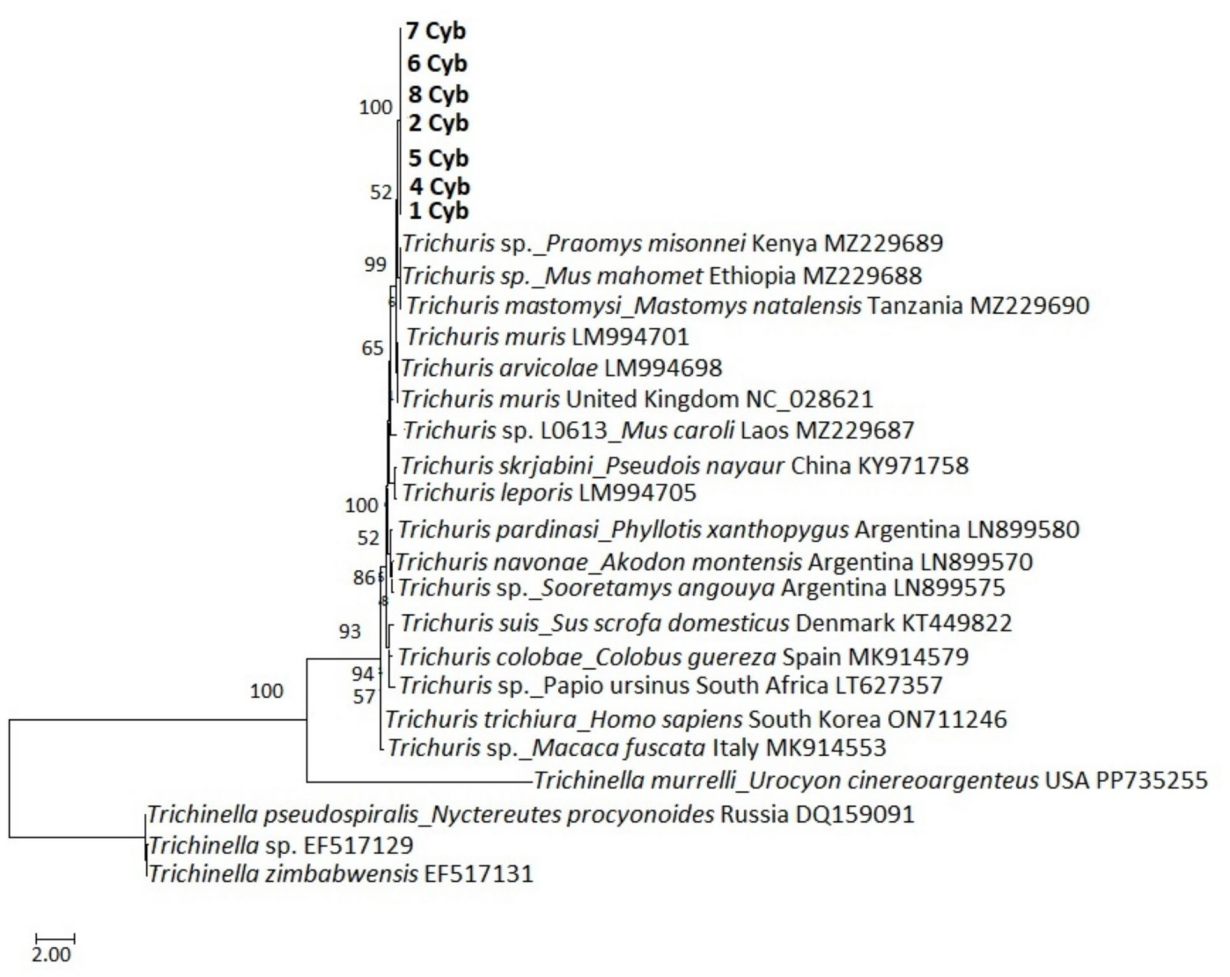
Phylogenetic analysis of *Trichuris* spp. cytochrome b sequences obtained in this study together with published sequences. The tree was generated using a maximum likelihood method with 1000 bootstrap replicates as implemented in the Molecular Evolutionary Genetics Analysis (MEGA) software version 11.0. Scores at the nodes represent corresponding bootstrap support. The scale bar is proportional to the genetic distance in terms of nucleotide substitutions per site. The cytochrome b sequences obtained in present study are highlighted in bold (1 Cytb to 8 Cytb). The tree was rooted using a *Trichinella pseudospiralis* sequence from raccoon dog (*Nyctereutes procyonoides*) (Accession number PP735255) in the USA, a *Trichinella* sp. sequence (accession number EF517129) and a *Trichinella zimbabwensis* (accession number EF517131). The *Trichuris* specimens were isolated from *M. natalensis* across a wildlife-human-domestic animal interface, in Mpumalanga Province, South Africa in October 2020

The genetic relationships between the new *Trichuris* sp. specimens and corresponding published sequences of the three regions or genes (ITS1, ITS2 and *cytb*) are shown in Figs. 4, 5, and 6, respectively. The obtained sequences were submitted to GenBank under accession numbers PQ600870-PQ600877 (ITS1), PQ583367-PQ583374 (ITS2) and PQ562420-PQ562426 (*cytb*) and consistently grouped in a separate clade from published sequences, suggesting a possible new lineage of *Trichuris* species in *Mastomys* rodents in South Africa. Assessment of evolutionary distances between the new sequences showed that they were identical within the ITS1 region and *cytb* region/gene, while the ITS2 region was identical in five sequences but showed slight variation in three sequences, with a 0.002 to 0.004 proportion of nucleotide differences between pairs of sequences (Table 3).

**Table 3:** Description of primers used during PCR analysis of *Trichuris* species recovered from *Mastomys natalensis* captured across a wildlife-human-domestic animal interface in Mpumalanga province, South Africa in October 2020

Target gene	Primers’ name and sequence (5′……..3′)	Amplicon length (bp)	Reference
ITS1	NC5: GTA GGT GAA CCT GCG GAA GGA TCA TT	῀500 bp	
	5.8SR: GAG TGT CAC GTC GTT CTT CA		(Rivero et al. 2020; Rivero et al. 2022)
ITS2	TrF: CTC GTA GGT CGT TGA AGA AC	῀500 bp	
	NC2: TTA GTT TCT TTT CCT CCG CT		
Cytochrome b	D769: GAG TAA TTT TTA TAA TRC GRG AAG T	῀570 bp	(Callejon et al. 2015; Rivero et al. 2022)
	D770: AAT TTT CAG GRT CTC TRC TTC AAT A		

## Discussion

The overall nematode prevalence was comparable between the two rodent species (19.1% in *M. natalensis* and 25.9% in *M. coucha*). However, the nematode species composition differed between the two rodents on descriptive analysis, with *Trichuris* sp. being more prevalent in *M. natalensis* compared to *M. coucha*, and *Abbreviata* sp. being more prevalent in *M. coucha* compared to *M. natalensis*. A previous study in South Africa recorded comparable prevalence values for *Trichuris muris* in *M. natalensis* (15.4%) and, in addition, recorded a higher infection in *M. natalensis* compared to *M. coucha* (7.1%) (Spickett et al. 2019a). In Tanzania, average infection levels of 23.3% (range 4.8–40.9%) were recorded for *Trichuris* spp. in *M. natalensis* at multiple localities (Ribas et al. 2013). A study conducted in Senegal recorded even higher infection levels (range between 25 and 67%) in *M. natalensis* (Brouat et al. 2007). In discordance with the present findings, Julius et al. (2018a) recorded a lower prevalence (1.2%) for an unknown *Trichuris* sp. in *M. coucha* in Gauteng, South Africa. In addition, it is interesting to note that whipworms were more consistently recorded in *M. natalensis* and at higher infection levels compared to other co-occurring rodent species in South Africa (Spickett et al. 2019a) and Tanzania (Ribas et al. 2013). However, further comparative studies are required to confirm the host association of *Trichuris* species.

The *Abbreviata* sp. in the current study was more predominant in *M. coucha*, with a prevalence of 22.2%, than 1.5% for *M. natalensis.* In South Africa, Spickett et al. (2017) recorded *Abbreviata* sp. with a prevalence of 0.7% in *Rhabdomys dilectus* and an absence in *R. pumilio* (Spickett et al. 2017). Further, the Spickett et al. (2019a) study in South Africa recorded a prevalence of 5.9%* Abbreviata* sp. in *Lemniscomys rosalia*, 5.7% in *Micaelamys namaquensis*, and 0.7% in *R. dilectus*; 0% prevalence was recorded for the *Mastomys* sp. Brouat et al. (2007) also found* Abbreviata* sp. but in another *Mastomys* species, *M. erythroleucus*, with an overall prevalence of 1.2% captured from different localities within Senegal, and none was recorded in *M. natalensis*. The abundance of the *Abbreviata* sp. is in low ranges (0–6%) in the above studies; however, more studies are needed to confirm the influence of host factors on the nematode prevalence.

The current study did not find a significant association between nematode abundances and habitat type. This is most probably due to low nematode infections. If higher infections were recorded, it is possible that nematode species composition might be related to habitat type. A recent study by Little et al. (2024) at the same localities recorded that *M. natalensis* occurred in the village and agricultural habitats while *M. coucha* was in the natural and agricultural habitats. It is thus possible that *Trichuris* sp. prefers anthropogenic habitats while *Abbreviata* sp. prefers natural habitats, and this could be linked to the difference in life cycles, with *Trichuris* sp. having a direct type while *Abbreviata* sp. has an indirect type, which requires an intermediate host (insect host) more likely to be found in natural habitats. A study in Kedougou, Southeastern Senegal (Brouat et al. 2007) reported *Trichuris muris* as the most prevalent nematode in dense villages as compared to the open savanna fields, with a prevalence of 38.5% in the commensal *M. natalensis* and 2.4% in semi-commensal *M. erytholeucus*, while *Abbreviata* sp. was only recorded in *M. erytholeucus* at 1.2% prevalence. In Tanzania, *Trichuris* spp. were prevalent in *M. natalensis* that were trapped in agricultural habitat (Ribas et al. 2013). However, a study conducted in the winter rain fall Fynbos region of South Africa recorded a *Trichuris* sp. at similar infection levels in *R. pumilio* in natural (1.4%) and urban (1.1%) habitats (Froeschke and Matthee 2014).

Despite our study recovering relatively few *Trichuris* specimens, the average total body length, anterior length, posterior length, and spicule base width for the male *Trichuri*s sp. are in similar ranges to those obtained by Feliu et al. (2000) for *T. muris* and *T. arvicolae* in Spain. The selected average characteristic measurements obtained for the females in this study were also in ranges of those reported by Feliu et al. (2000) in which the mean vaginal length (0.15 mm) varied greatly from *T. muris* (0.45–1.11 mm), but was slightly different from *T. arvicolae* (0.20–0.70 mm). The mean spicule length (0.83 mm) was consistent with that of *T. mastomysi, T. carlieri* s.l. recovered from *Gerbilliscus vicinus*, and that of *T. muris* (0.58–0.99 mm) recovered from *Mus domesticus* in Tanzania (Ribas et al. 2013). The posterior-anterior length ratio recorded for males was 1:1.98, which is slightly higher than the one reported for clade one of West African *Trichuris* sp. (1:1.29–1:1.39) and (1:1.48–1:1.50) for clade three (Ribas et al. 2017), while the average egg length (0.061 mm) and width (0.030 mm) were slightly higher than those reported for the *Trichuris* sp. in West Africa (egg length of 0.050 mm and an average of 0.026–0.030 mm egg width) (Ribas et al. 2017). The morphometric comparison of African female *Trichuris* sp. remains scanty, and these findings contribute to the reference literature. The present findings clearly indicate that the species diversity of *Trichuris* cannot be confirmed by only conventional morphometric methods, which lack diagnostic specificity, as observed in previous studies (Feliu et al. 2000; Liu et al. 2012; Ribas et al. 2013; Cavallero et al. 2015).

The ITS1, ITS2, and *cytb* sequencing results showed that the *Trichuris* sp. recovered in the study belongs to a distinct genetic clade with relatively high bootstrap values. The ITS1 sequences formed a separate clade that was relatively close to a clade that comprises *Trichuris* sp. sequences from *M*. *natalensis* in Guinea, *Trichuris* sp. sequences from *M*. *huberti* in Senegal, and *T*. *carlieri* sequences from *G*. *vicinus* in Tanzania*.* For the ITS2 region, the sequences formed a separate clade that was relatively close to a group with *T*. *mastomysi* from *M*. *natalensis* in Tanzania and *T. carlieri* s.l from *G. vicinus* in Maguha, Tanzania (Ribas et al. 2013). The observed clade and sequence differences indicate a variation in the *Trichuris* sp. recovered in the present study from those reported in other parts of Africa and globally and present a new lineage within the genus *Trichuris* populations in the Mnisi community area. These findings contribute to our understanding of nematode diversity and evolution in this geographical region. They also confirm the adaptable nature of *Trichuris* sp. and the ability of the genus to infect various confamilial host species.

Although this study focused on one province, the study site being a human-domestic-wildlife interface that includes fairly large rural areas bordering several large nature reserves in the Mpumalanga Province, South Africa, offers a unique platform to study parasite diversity across various anthropogenic habitat types (agriculture and village). *Mastomys* rodents are semi-commensals and hence they move in all habitats (crop, natural reserve, and households); so, these samples would inform the nematode diversity for the particular season of rodent capture. This is the first study of its kind in South Africa. Only 15 *Trichuris* species specimens were recovered for morphometry analysis; however, identification of the specimens was conducted in consultation with experienced helminthologists at the National Helminth Reference Collection. It can be difficult to differentiate between species in the genus *Trichuris* using morphological characteristics alone (Ribas et al. 2013) and therefore, morphological features in the study were mainly used as a first-line identification step to ensure DNA is only extracted from specimens primarily identified as *Trichuris* for more specific molecular identification or phylogenetic analysis. Only three mitochondrial genes were assessed, and we used *cytb* gene instead of cytochrome c oxidase subunit I (CO1), which, although may have implications in confirming the novelty of the *Trichuris* sp., a number of studies have demonstrated that *cytb* can be used as an alternative mitochondrial gene for the same purpose of molecular identification at the species level. We prioritised *Trichuris* and not *Abbreviata* (the other nematode found) based on the multi-host nature of the genus *Trichuris* and possible zoonotic potential of the genus.

## Conclusions

*Trichuris* sp. and *Abbreviata* sp. were recorded in *M. natalensis* and *M. coucha* across a wildlife-human-domestic-animal interface in the savanna biome. Although infections were generally low for statistical analysis, the two nematode species seem to exhibit distinct habitat preferences, with a higher incidence of *Trichuris* sp. in anthropogenic habitats in contrast to a higher incidence of *Abbreviata* sp. in natural habitat. The sequences obtained from the unidentified *Trichuris* sp. formed a unique phylogenetic lineage, and this necessitates further studies for confirmation of a possible new species. This study emphasizes the importance of using an integrated approach of morphological measurements and molecular analysis to accurately categorize *Trichuris* spp. There is a need for future studies that involve a larger sample size of *Mastomys* species together with the inclusion of other sympatric rodent species across multiple habitat types.

## Supplementary Information

 Below is the link to the electronic supplementary material.

ESM 1 (DOCX 4.62 MB)

## Data Availability

All relevant data generated or analysed during this study are available upon reasonable request from the corresponding author. The genetic sequences of the *Trichuris* species analysed in this study have been submitted to GenBank under accession numbers: PQ600870–PQ600877. These sequences include the ITS1, ITS2, and cytochrome b (*cytb*) gene regions.
