# Supplementary material for: Nematode diversity in Mastomys rodents (Rodentia: Muridae) across a wildlife-human/domestic animal interface and molecular characterization of Trichuris species from M. natalensis
Source: Parasitol Res. 2025 Jun 11;124(6):64. doi: 10.1007/s00436-025-08507-y (PMC12152047; doi:10.1007/s00436-025-08507-y)
Supplement: Supplementary file 1 — (DOCX 4.62 MB) [file 436_2025_8507_MOESM1_ESM.docx]

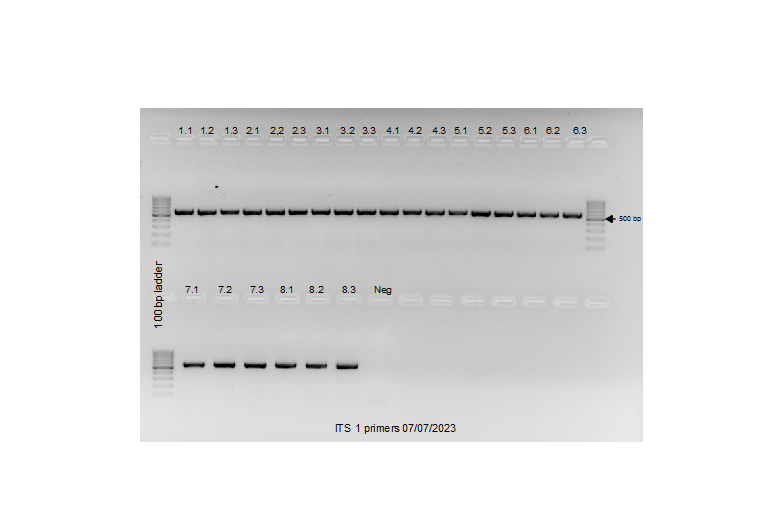


Fig 1: Showing gel and blot image of uncropped ITS1 gene done in triplicates for the 8 *Trichuris* sp. samples


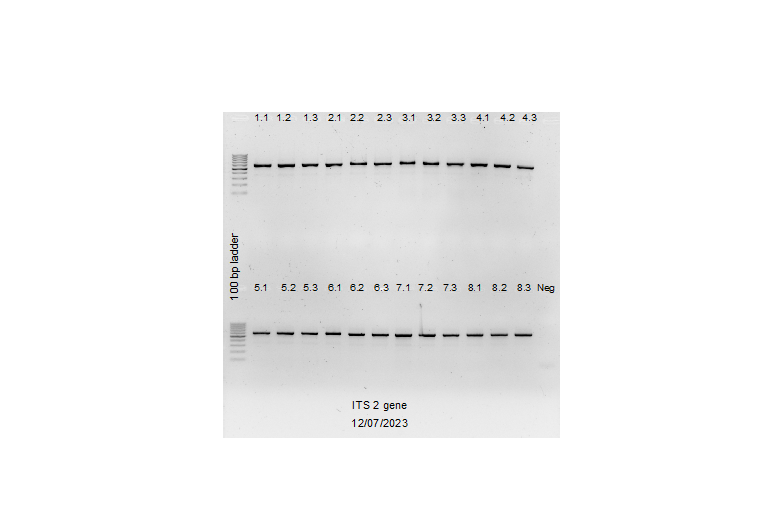


Fig2: Showing gel and blot image of uncropped ITS2 gene done in triplicates for the 8 *Trichuris* sp. samples


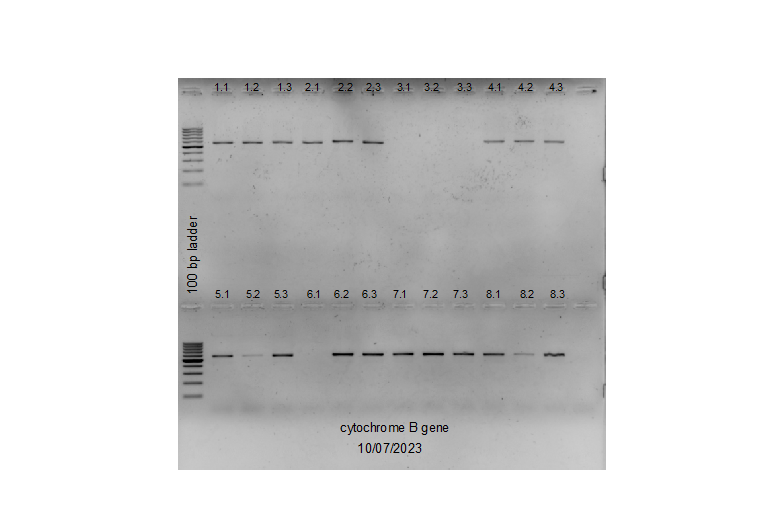


Fig3: Showing gel and blot image of uncropped cytb gene done in triplicates for the 8 *Trichuris* sp. samples
